# A pathophysiological model of gait captures the details of the impairment of pace/rhythm, variability and asymmetry in Parkinsonian patients at distinct stages of the disease

**DOI:** 10.1038/s41598-021-00543-9

**Published:** 2021-10-27

**Authors:** Marco Godi, Ilaria Arcolin, Marica Giardini, Stefano Corna, Marco Schieppati

**Affiliations:** 1 Division of Physical Medicine and Rehabilitation, Scientific Institute of Veruno, Istituti Clinici Scientifici Maugeri IRCCS, 28010 Gattico-Veruno, NO Italy; 2 Scientific Institute of Pavia, Istituti Clinici Scientifici Maugeri IRCCS, 27100 Pavia, Italy

**Keywords:** Physical examination, Rehabilitation, Disability, Parkinson's disease

## Abstract

Locomotion in people with Parkinson’ disease (pwPD) worsens with the progression of disease, affecting independence and quality of life. At present, clinical practice guidelines recommend a basic evaluation of gait, even though the variables (gait speed, cadence, step length) may not be satisfactory for assessing the evolution of locomotion over the course of the disease. Collecting variables into factors of a conceptual model enhances the clinical assessment of disease severity. Our aim is to evaluate if factors highlight gait differences between pwPD and healthy subjects (HS) and do it at earlier stages of disease compared to single variables. Gait characteristics of 298 pwPD and 84 HS able to walk without assistance were assessed using a baropodometric walkway (GAITRite®). According to the structure of a model previously validated in pwPD, eight spatiotemporal variables were grouped in three factors: pace/rhythm, variability and asymmetry. The model, created from the combination of three factor scores, proved to outperform the single variables or the factors in discriminating pwPD from HS. When considering the pwPD split into the different Hoehn and Yahr (H&Y) stages, the spatiotemporal variables, factor scores and the model showed that multiple impairments of gait appear at H&Y stage 2.5, with the greatest difference from HS at stage 4. A contrasting behavior was found for the asymmetry variables and factor, which showed differences from the HS already in the early stages of PD. Our findings support the use of factor scores and of the model with respect to the single variables in gait staging in PD.

## Introduction

Unpredictable appearance of signs of the disease, early or late onset time, hesitations in the diagnosis, current treatments with drugs or physical therapy, subjective evaluations performed in the clinical setting and lack of consistency in gait features make the interpretation of gait findings in patients with Parkinson’s disease (pwPD) unclear and difficult to classify^1–6^. Among the different spatiotemporal gait variables, speed is the gold standard in gait assessment because it is relatively easy to measure and normative data are readily available^7–10^. Some studies have advocated gait speed as the best expression of locomotor control, combining the influence of alterations or adaptations occurring anywhere in the brain locomotor centres^11,12^. In pwPD, gait speed is reduced^10,13–15^, is linked with falls^16^ and with impaired quality of life^17^, and is highly sensitive to ageing^18^.

However, gait speed alone does not explain the underlying spatiotemporal gait abnormalities. In addition to reduced gait speed, pwPD typically have smaller stride length, longer double support time and increased variability and asymmetry^4,10,19,20^. While some variables show clear differences with respect to matched controls^21^, there is little agreement about gait variability and asymmetry^4,21^. To establish whether and how the differences between pwPD and healthy subjects (HS) evolve as the disease progresses is not an easy task^22^. Previous investigations showed increased gait variability to be associated with duration and severity of the disease^23,24^. In a recent prospective study, gait variability showed a linear progression over a 5-year observation period^25^. Studies have also shown increased spatial/temporal gait asymmetry in pwPD^26,27^, especially in the off-levodopa state^28^, although findings of gait asymmetry in the on-medication state are inconsistent^29^.

Quantifying gait and balance deficits is important for monitoring patients over time, for adjusting medication and for the management of fall risk^30^. At present, evaluation of gait is supported by subjective tools such as qualitative clinical assessments^30,31^, even though a change in a single variable (e.g. gait speed) may not be disease-specific or suitable for assessing the evolution of locomotion pattern over the course of the disease. Conceptual models of gait have been proposed in the past and continue to receive attention. By identifying a minimal set of significant and unrelated variables and grouping them into factors or domains, models can provide a framework for assessing the main gait characteristics^32–34^. Hypothesis-driven research to identify the main contributory elements to gait disturbance will improve the clinical interpretation of gait problems^1,12^. Such models have been created and used for evaluating the gait features of elderly subjects, and of patients with various diseases such as PD, hip fracture, traumatic brain injury or mild cognitive impairment^1,33–40^.

The independent factors emerging from the models have the declared drawback of being the result of combining distinct variables. Therefore, the appropriate selection of the variables at the origin of the model design is crucial. On the other hand, the factors represent a synthetic description of key domains of gait (e.g., pace, rhythm, asymmetry, variability). Although gait models should be based on independent domains of gait, they can contain redundancy. Some published models^1,33,36^ feature highly inter-related (i.e., step time and cadence) or less reliable variables (i.e., some variability variables expressed as standard deviation (SD)), or variables that, depending on the study considered, were dispersed over several domains (i.e., step time variability variable). To address these limitations, a conceptual model of gait has been recently proposed by Arcolin et al.^38^, which places reliance on carefully selected gait variables. Composed of three independent primary factors (pace/rhythm, variability, and asymmetry), the model has withstood a confirmatory validation and has shown a good level of structural validity for assessing gait in pwPD^41^. Of note, the model has also been replicated and confirmed in HS^42^, and hence is a valid instrument for assessing gait in both pwPD and HS.

To date, however, doubt remains whether collecting variables into factors will enhance interpretability and be useful in the clinical assessment of disease severity. Until now, the gait variables identified in the models have been considered in isolation, without using a cumulative score of the factors (made of groups of variables). Moreover, even if it is known that most gait variables are affected in the more advanced stages of PD, it has not been established whether and how gait, assessed with gait variables or factors, evolves with the progression of PD severity, in particular in the initial to intermediate stages of disease. In this study, we compared gait between HS and pwPD using the model of Arcolin et al.^38^. While HS were always considered as entire sample, in the first part of the study pwPD were analysed as an all-inclusive sample, whereas in the second part they were subdivided into the different stages of severity according to the modified Hoehn and Yahr (H&Y) scale^43^. Our questions were: (1) which variables best underline the differences between HS and pwPD? (2) Does the accuracy in distinguishing PD from HS increase by grouping the variables into factors and into a model? (3) Compared to HS, at which clinical stage do pwPD begin to show differences from HS in locomotion? (4) Can factors and the overall model highlight gait differences between pwPD and HS at an earlier stage of disease compared to the single variables?

## Methods

### Participants

All participants were recruited from the Posture and Movement Laboratory at the Scientific Institute of Veruno (Novara, Italy) of the Istituti Clinici Scientifici Maugeri (IRCCS), between February 2014 and September 2019. Inclusion criteria for patients were a diagnosis of Parkinson’s disease, as defined by the UK Parkinson’s Disease Society Brain Bank Criteria^44^ and the ability to walk independently without assistance, even if the use of a walking aid (walker or cane) was accepted. Disease severity was quantified using the modified H&Y scale^43^, which contains two added stages: 1.5 and 2.5^45^. HS were generally recruited among patients’ relatives and considered for the study if they were aged 50–80 years (see Table 1). The study was performed according to the Declaration of Helsinki. The Ethics Committee of ICS Maugeri SpA SB approved this cross-sectional study (approval number #905 CEC) and all participants gave their informed consent.

**Table 1:** Clinical characteristics of patients with Parkinson’s Disease (pwPD) and healthy subjects (HS).

Characteristics	pwPD (N = 298)	HS (N = 84)	p value
Gender (n)	159M; 139 F	40M; 44 F	0.86
Age (years)	70.0 (7.8)	71.5 (8.2)	0.16
BMI (kg/m^2^)	26.1 (4.2)	25.1 (3.2)	0.06
MMSE (score)	26.9 (3.3)	28.3 (1.7)	0.07
Disease duration (years)	7.6 (5.4)	–	–
LEDD (mg)	793.3 (501.0)	–	–
H&Y stage—mean	2.5 (0.6)	–	–
H&Y 1 (n)	7	–	–
H&Y 1.5 (n)	27	–	–
H&Y 2 (n)	60	–	–
H&Y 2.5 (n)	99	–	–
H&Y 3 (n)	93	–	–
H&Y 4 (n)	12	–	–

BMI, body mass index; MMSE, mini-mental state examination; LEDD, levodopa equivalent daily dosage; H&Y, modified Hoehn and Yahr scale.

PwPD and HS were excluded if they had neurological disorders (e.g., traumatic brain injury, multiple sclerosis or polyneuropathy), recent orthopaedic injuries or surgery, pain or impairments that could interfere with balance and mobility, severe musculoskeletal problems affecting the lower extremity or spine, cognitive problems (i.e. Mini-Mental State Examination, MMSE, < 24) or were unable to follow instructions. PwPD at H&Y stage 5 (‘wheelchair bound or bedridden unless aided’) were excluded. At the time of assessment, pwPD were pharmacologically treated (Table 1) and in on-medication state; if the patient began to feel stiff or blocked, the test was stopped and the trial was eliminated.

### Gait protocol and analysis of spatiotemporal data

Spatiotemporal gait variables of HS and pwPD were acquired using an electronic walkway (GAITRite^®^, CIR Systems, Sparta, NJ, USA)^46^. Participants were asked to walk barefoot on the walkway at a comfortable speed with the standard instruction “walk along the walkway at your normal speed”. The walk started 2 m before the 4.60 m walkway, to compensate for problems with gait initiation^47^, and stopped 2 m after the walkway to compensate for speeding up/slowing down at the end. During the trials, patients were allowed to use their usual walking aid, if they needed it. Subjects repeated the trial four times^48^. Since the gait variables of the first trial differed significantly from the other three, the first trial was considered as a familiarisation trial and not included in the analysis^38^. Therefore, we considered only the 2nd, 3rd and 4th trials, with a total of 21–24 steps. This ensured a good inter-trial reliability^38,49^.

### Data processing

Based on the recorded footfalls, for each walking trial the GAITRite^®^ walkway automatically calculates 46 gait variables, of which 36 separately for each foot (making a total of 82 variables). First of all, the values of the gait variables for the 2nd, 3rd and 4th trials were averaged and the resulting combined measures were used for the following analysis. Since we decided to reproduce the model of Arcolin et al.^38^, we a priori selected the eight variables here included, which were: gait speed, step time (time in seconds elapsed from the first contact of one foot to the first contact of the opposite foot), double support time, step length coefficient of variance (CV), swing time CV, step velocity CV, step time asymmetry and swing time asymmetry. All these gait variables proved to have an excellent reliability, with values of interclass correlation coefficients between 0.75 and 0.99^38^. Variability was expressed as CV (CV = SD/mean × 100, where “mean” is the average value of each spatiotemporal variable for each patient). Measures of asymmetry were calculated as 100 ×|ln(left/right)|, where “left” represents the left foot average and “right” the right foot average for both asymmetry variables (step time and swing time), and the symbol “|…|” means absolute value, necessary for obtaining positive asymmetry values since we always considered the natural logarithm of left/right instead of the ratio longer-step/shorter-step^26^.

### Construction of the gait model and factor scores

The model was originally confirmed in a sample of pwPD^38^ and subsequently applied in a sample of HS^42^. In this study, we used the model in both cohorts to verify its discriminating power in identifying the progression of gait impairment over the different H&Y stages with respect to HS. In particular, the eight gait variables from both pwPD and HS, selected a priori from the model of Arcolin et al.^38^, were used and a new exploratory factor analysis (EFA), which is necessary for creating the model, was conducted using the methods previously reported^38^. After determining the number of factors to be considered, the variables were distributed into factors based on the EFA matrix. This represents the correlations between the variables and factors: correlation coefficients ≥ 0.40 were considered relevant for a given factor^50^. By replicating the EFA of Arcolin et al.^38^ and using the spatio-temporal variables of both pwPD and HS, we expected to allocate the eight gait variables into three factors (pace/rhythm, variability and asymmetry). This permitted the generation of the three factor scores and of the model, the comparison between HS and the overall sample of pwPD, and between HS and the pwPD at the different H&Y stages. After EFA, we computed the factor scores, one for each factor of the gait model. Using the weighted sum score (WSS) method, factor scores were calculated by transforming the eight gait variables into z-scores and then aggregating them based on the EFA results (for a detailed description see the Supplementary Material)^51^. Higher scores indicate greater gait impairment. In order to combine the three factor scores (pace/rhythm, variability and asymmetry) into an overall score of the full gait model, we performed a logistic regression analysis (see below).

### Comparison of pwPD with HS

We assessed the ability of each gait variable, factor score and score of the all-inclusive model to correctly detect subjects belonging to each of the two cohorts (pwPD and HS) through Receiver Operating Characteristic (ROC) curves. We used the area under the ROC curve (AUC) and its 95% Confidence Interval (95% CI) to assess the diagnostic performance of each variable and factor and the model. The AUC ranges between 0.5 (no diagnostic accuracy beyond chance) to 1.0 (perfect diagnostic accuracy): an area > 0.90 indicates high accuracy, 0.70–0.90 moderate accuracy, 0.50–0.70 low accuracy and < 0.50 a chance result^52^. To describe the accuracy of the ROC curve, we also reported the sensitivity and specificity of the cutoff values at which the pair of sensitivity and specificity values was maximised, hence the point that maximises the discrimination between true-positive rate and false-positive rate. In fact, sensitivity is the number of true positive decisions/the number of actually positive cases, while specificity is the number of true negative decisions/the number of actually negative cases. We then calculated the accuracy, which is the proportion of subjects correctly classified at that cutoff (both true positives and true negatives) to the total number of subjects.

### Comparison between HS and pwPD at different H&Y stages

To determine the sensitivity of each variable, factor or of the overall model in assessing the difference between the pwPD at the different H&Y stages and the HS, we used the Cohen’s effect size (ES)^53^. It is estimated that ES = (μ1 − μ2)/σ, where μ1 and μ2 are the means of the groups compared (in this case, each gait variable at each stage of pwPD vs. HS) and σ is the pooled SD of both means. Values of ES < 0.2 represent no differences, from 0.2 to 0.49 small differences, from 0.5 to 0.79 medium differences, from 0.8 to 1.19 large, from 1.2 to 1.99 very large and ≥ 2.0 huge differences^53,54^. Seventy-two ESs ((8 variables, 3 factors, 1 model) × (6 stages of the scale)) were obtained.

### Statistical analysis

Normality of data distribution for each gait measure (i.e., variable, factor, model) was determined by the Shapiro–Wilk test. To detect differences between the clinical characteristics of HS and pwPD, non-paired Student’s *t*-tests were performed for age, body weight, height and body mass index (BMI), while the Mann–Whitney U test was used for the MMSE. The categorical variable (gender) was tested using the chi-squared (χ^2^) test. Where not specified, text and tables report the results as mean ± SD, while the Figures report the mean ± standard error (SE). The differences in the factor scores between HS and pwPD at the various H&Y stages were assessed by one-way analysis of variance (ANOVA). When ANOVA gave a significant result (p < 0.05), the post hoc Fisher’s Least Significant Difference test was used to assess differences between the variables evaluated for each group and the Bonferroni correction was applied.

A logistic regression analysis was performed to provide the score of the model. The STATA command “logit” was used with the factor scores of pace/rhythm, variability and asymmetry as independent variables and the condition (HS or PD) as dependent variable. We used the “roctab” command of STATA to obtain the non-parametric estimation of the ROC curve and the AUC values for gait variables and factor scores. Conversely, for the model of gait we used the “lroc” command after logistic regression analysis. AUCs were compared with the STATA command “roccomp” and χ^2^ test was used to calculate the statistical difference between AUCs. All statistical analyses were performed using the STATA R13.0 software package.

## Results

### The two cohorts: pwPD and HS

**Figure 1:**
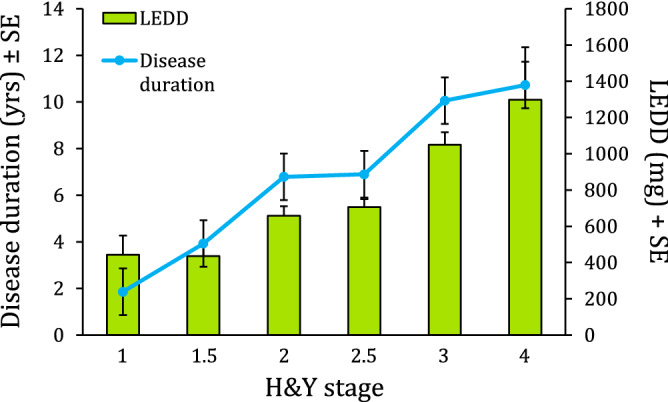
Disease duration (left ordinate) and levodopa equivalent daily dosage (LEDD) (right ordinate) over the different stages of the H&Y scale (abscissa). The green bars and the blue line show the parallel trend of disease duration and LEDD. The H&Y staging faithfully reflected the progression of the disease and the amount of medication administered.

Of 359 pwPD screened for eligibility, 61 (17%) did not meet the inclusion criteria and were excluded. The remaining 298 (139 women and 159 men, mean age 70.0 years) formed the pwPD cohort (Table 1). Stratified by the modified H&Y stage of severity, patients were classified at: stage 1, n = 7; stage 1.5, n = 27; stage 2, n = 60; stage 2.5, n = 99; stage 3, n = 93; stage 4, n = 12. Disease duration ranged from 6 months to 26 years, with a mean of 7.6 years, and correlated with the levodopa equivalent daily dosage (r = 0.58, p < 0.0005) (Fig. 1). Ninety-one participants commonly used a walking aid in daily life, but only 42 of these (walker, n = 20; cane, n = 22) made use of it during gait assessment on the baropodometric walkway. Of these, 12 were at H&Y stage 2.5, 23 at stage 3 and 7 at stage 4.

Regarding the HS group, 96 healthy subjects were screened for eligibility, of which 12 (13%) not meeting the inclusion criteria were excluded, leaving 84 participants (44 women and 40 men, mean age 71.5 years), who constituted the HS cohort. Clinical characteristics of pwPD and HS are reported in Table 1. No significant differences were found between groups for gender, age and body mass index. The score of MMSE was slightly lower in pwPD than in HS.

### Discriminating power of gait variables

Table 2 shows the mean values of the eight variables for both the pwPD and the HS cohorts. All gait variables differed significantly between HS and the overall sample of pwPD. PwPD had a reduced gait speed and longer step time and double support time compared to HS. The mean value of the double support time was 32% and 21% of the gait cycle in pwPD and HS, respectively. Variability and asymmetry variables were greater in pwPD than in HS.

**Table 2:** Mean values of the eight gait variables (± SD) in the patients with Parkinson’s Disease (pwPD) and healthy subjects (HS).

Variable	pwPD (N = 298)	HS (N = 84)	p value
Gait speed (cm/s)	100.05 (30.42)	122.29 (17.7)	< 0.0005
Step time (s)	0.54 (0.10)	0.51 (0.05)	< 0.05
Double support time (s)	0.32 (0.15)	0.26 (0.05)	< 0.005
Step length CV (%)	4.73 (3.10)	2.89 (1.10)	< 0.0005
Swing time CV (%)	5.68 (2.91)	4.00 (1.66)	< 0.0005
Step velocity CV (%)	4.37 (2.89)	2.95 (1.24)	< 0.0005
Step time asymmetry (%)	4.27 (4.58)	2.16 (1.97)	< 0.0005
Swing time asymmetry (%)	5.22 (5.56)	2.65 (2.30)	< 0.0005

The ability of the gait variables, separately considered, to discriminate between pwPD and HS was assessed by ROC analysis (Table 3). Three variables showed moderate ability to discriminate between pwPD and HS: gait speed, step length CV and swing time CV. The remaining 5 variables (step time, double support time, step velocity CV, step time asymmetry and swing time asymmetry) had AUC values between 0.50 and 0.70, corresponding to low discriminative accuracy. Of note, gait speed, step length CV and swing time CV had the highest AUC value (0.73, 0.72 and 0.70, respectively), with similar accuracy in correctly classifying pwPD from HS (around 67%).

**Table 3:** Area under the curve (AUC) of the discriminating power of the eight gait variables, of the three factor scores and of the model. AUC values are reported for each measure, together with 95% confidence intervals (95% CI), sensitivity, specificity and accuracy.

Measure	AUC	95% CI	Sensitivity (%)	Specificity (%)	Accuracy (%)
Gait speed	0.73	0.67–0.78	75.3	65.3	67.4
Step time	0.55	0.49–0.62	57.3	48.1	55.7
Double support time	0.64	0.57–0.70	56.4	63.6	57.9
Step length CV	0.72	0.66–0.78	65.0	75.3	67.1
Swing time CV	0.70	0.63–0.76	66.2	67.5	66.5
Step velocity CV	0.67	0.61–0.73	59.9	62.3	60.4
Step time asymmetry	0.66	0.60–0.73	63.9	67.5	64.7
Swing time asymmetry	0.66	0.60–0.72	64.6	62.3	64.1
Pace/rhythm	0.65	0.59–0.71	59.1	59.7	59.2
Variability	0.77	0.72–0.82	69.0	71.4	69.5
Asymmetry	0.66	0.60–0.73	63.9	67.5	64.7
Model of gait	0.81	0.76–0.86	72.8	72.7	72.8

### Exploratory factor analysis (EFA) of the model and ability of factor scores to discriminate between pwPD and HS

The EFA results on the correlation of each variable with the three factors are shown in the Supplementary Material. Despite our heterogeneous sample (containing both HS and pwPD), the distribution of variables of both cohorts into factors was the same as for the model presented by Arcolin et al.^38^. The pace/rhythm factor included three variables (gait speed, step time, and double support time), the variability factor four variables (step velocity CV, step length CV, swing time CV and gait speed) while the asymmetry factor contained two variables (step time asymmetry and swing time asymmetry) (see Supplementary Material).

**Figure 2:**
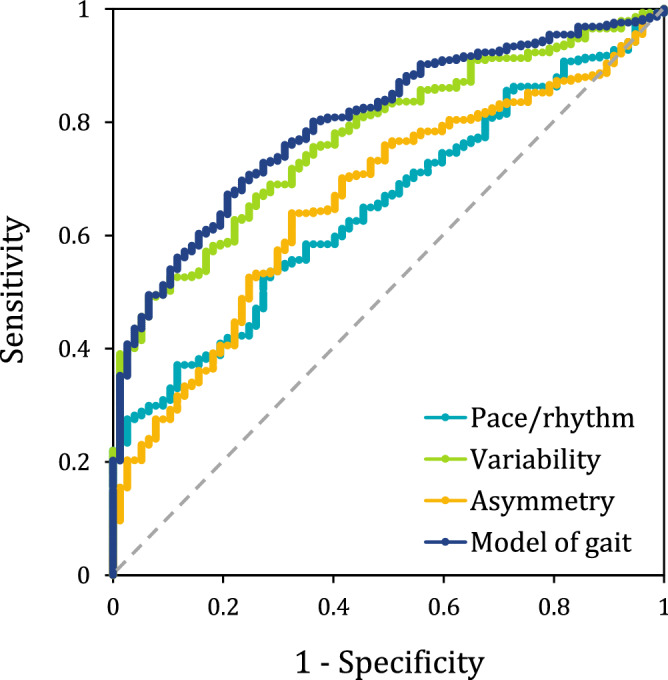
Receiver Operating Characteristic (ROC) curves assessing the ability of the three factor scores and of the overall gait model to discriminate between pwPD and HS. The light-blue line represents the pace/rhythm factor, the green line represents the variability factor, the yellow line represents the asymmetry factor, while the dark blue line represents the gait model with the three factor scores collapsed together. The grey dotted line represents the random classifier (“no discriminated” line), where the AUC is equal to 0.50.

The three factor scores in isolation, created for each of the three identified factors (see Supplementary Material), showed a low to moderate ability to discriminate between pwPD and HS (Fig. 2, Table 3). The pace/rhythm and the asymmetry factors had a low ability to correctly classify pwPD and HS (AUC = 0.65 and AUC = 0.66, respectively), while the variability factor showed the highest ability to discriminate between pwPD and HS (AUC = 0.77). The logistic regression model, created by combining the three factor scores, proved to have a good ability to discriminate pwPD from HS (Fig. 2, Table 3). The AUC of the model (= 0.81) was larger with respect to the AUCs of each single factor in isolation (for pace/rhythm and asymmetry, p < 0.0005; for variability, p < 0.05).

### Variables and factor scores split by H&Y stage in comparison to HS

Variables, factor scores and the model, after evaluating their ability to discriminate HS from the overall cohort of pwPD, were then evaluated for each stage of pwPD severity (Supplementary Fig. S1). The largest difference in the scores of the three factors was found between HS and pwPD at stages 2.5, 3 and 4, in spite of the obvious larger variance in stage 4 compared to the others.

**Figure 3:**
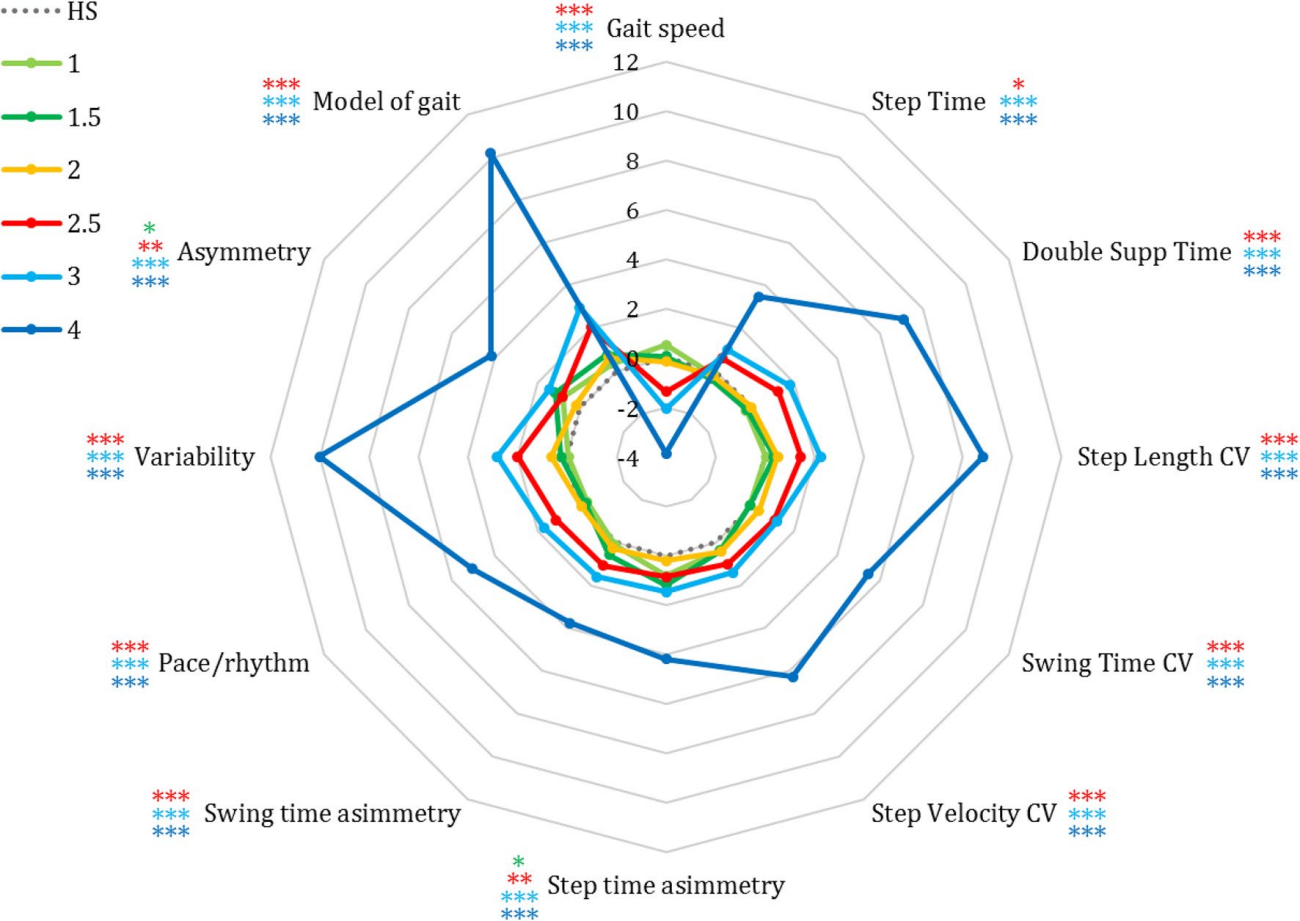
Radar plot illustrating the z-scores of pwPD and HS for the 8 gait variables, 3 factor scores and overall gait model. The grey dashed line passing through zero represents the z-scores data of HS, while the other coloured lines represent the z-scores data of the six H&Y stages of pwPD. The values on the axis indicate how often the z-scores for each variable, factor or model obtained by pwPD at the different H&Y stages differ from the corresponding data of HS. Coloured asterisks represent significant differences between each gait variables, factor scores and overall gait model of HS compared to those of pwPD at different stages: *p < 0.05; **p < 0.005; ***p < 0.0005. In particular, the asterisks coloured in dark green, red, light-blue and dark blue represent the differences of HS with pwPD at stages 1.5, 2.5, 3 and 4, respectively.

A radar chart was used to represent the evolution of the gait variables across the six stages of H&Y (Fig. 3). In order to compare pwPD and HS, the data were transformed into z-scores based on the means and SDs of HS (e.g., z-score pace/rhythm factor = (pace/rhythm factor of pwPD − mean pace/rhythm factor of HS)/SD pace/rhythm factor of HS). The chart shows that much as for the factor scores mentioned before, major differences with respect to HS emerged for pwPD at or above H&Y stage 2.5, except for step time asymmetry and asymmetry factor, which differed from HS also at H&Y stage 1.5 (p < 0.05). While for some variables and factors the differences with respect to HS increased gradually with the progression of H&Y stages, differences in double support time, step length variability and velocity, variability factor and the model increased disproportionately. The gait speed was the only variable which showed a different behaviour. While the other variables and factor scores increased with the progression of PD, at H&Y stages 2.5, 3 and 4 patients walk slowly. Hence, as the reference value (zero in the radar chart, corresponding to the dotted circle) is HS, the increase of the other variables and factor scores was expressed by positive values. On the contrary, the z-score of gait speed of pwPD become negative at H&Y stage 2.5 and higher.

### Gait impairment progression across H&Y stages in comparison to HS

**Figure 4:**
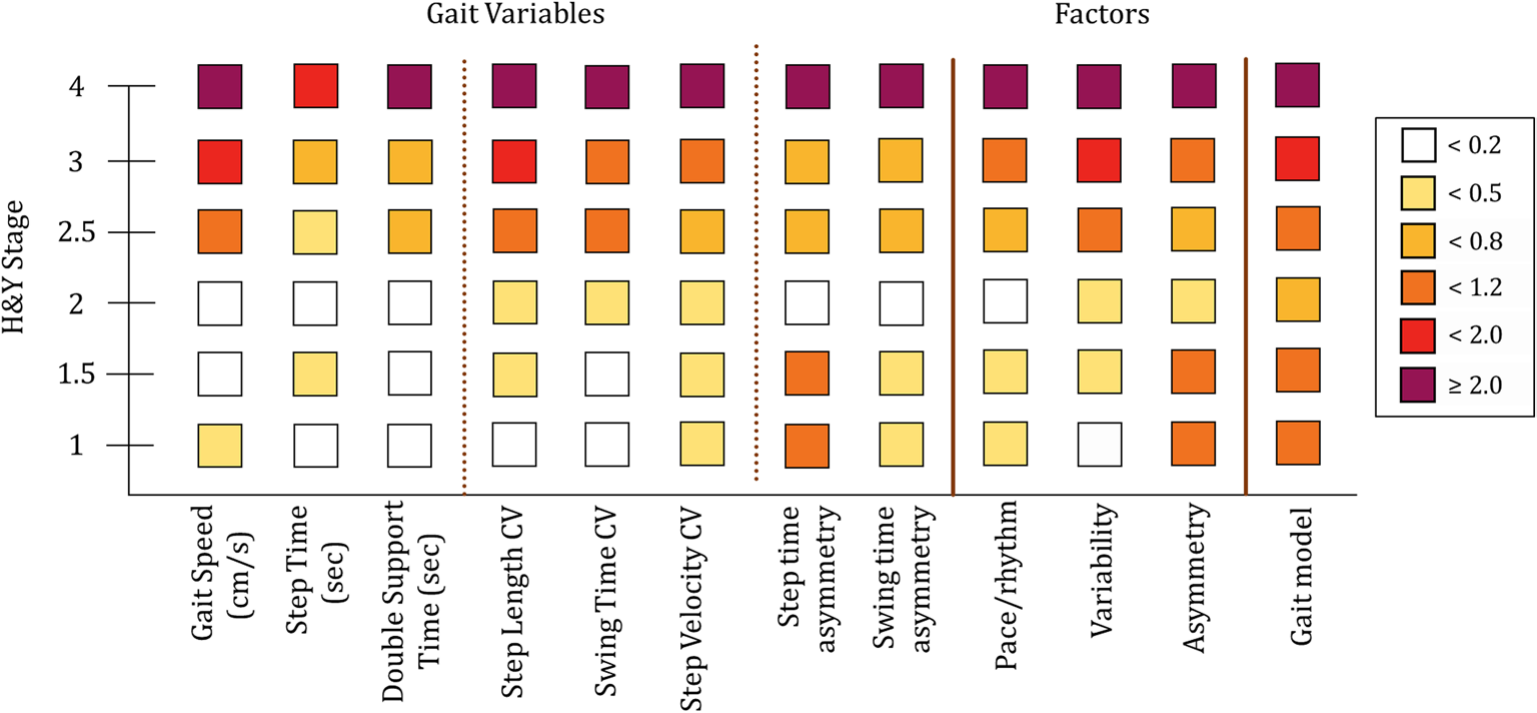
Effect sizes (ES) for the differences between pwPD (at each H&Y stage) and HS for each of the 8 gait variables, 3 factor scores and the gait model (abscissa). The magnitude of each ES for each stage of the H&Y scale (ordinate) is marked in colours according to the palette shown on the right: dark red > 2, huge effect; red > 1.2, very large effect; orange > 0.80, large effect; yellow 0.50–0.79, medium effect; sand 0.20–0.49, small effect; white < 0.20, no effect.

The effect size (ES) of the differences between pwPD and HS for the 8 gait variables, 3 factors and the gait model are shown in Fig. 4. All variables were sensitive to gait impairment in PD. The pace/rhythm variables (first three columns on the left) showed an increased amplitude of the differences between pwPD and HS as PD severity increased (from bottom to top of the panel). The values of the three variability parameters became worse as the disease progressed. The difference between HS and pwPD became explicit from stage 2.5 onwards, with the ES showing the highest value at stage 4. Small differences emerged also in the first stages in step length and step velocity CV. The two asymmetry variables were moderately different between pwPD and HS already at the first two stages of the modified H&Y scale. The differences were non-significant at stage 2 but then progressively increased in the higher stages. The ES of the differences for each of the three factors was broadly similar to that of the single constituent variables. Finally, the ES of the differences (pwPD vs. HS) for the model ranged from large to huge as H&Y stage increased. The combination of the three factor scores in the model detected a strong difference in gait features between HS and pwPD at all stages of the modified H&Y scale.

By scanning Fig. 4 from left to right, one can see that pwPD at stages 1 and 1.5 showed just a light to moderate change with respect to HS in the variability and asymmetry variables and for the pace/rhythm and variability factors, whereas the step time asymmetry and the asymmetry factor obviously highlighted the weight of asymmetry in the these initial PD stages, with large differences with respect to HS at stages 1 and 1.5. At H&Y stage 2, pwPD began showing an initial impairment in gait speed, but overall were not much different from pwPD at stages 1 and 1.5, except, remarkably, for the gait asymmetry variables and factor. It seems that larger differences start appearing at stage 2.5 and higher, becoming huge at stage 4.

## Discussion

This study has compared the gait characteristics of pwPD with those of HS and identified the gait changes in relation to the disease progression, focusing not only on the single gait variables, but also on factors (groups of variables) and on a model incorporating the distinct factors of gait. Our results show that the model applied here, based on non-redundant variables, can be used with confidence in the clinical setting as a reliable marker of gait quality in PD. One can also use the model to classify the disease as it progresses, matching the patient’s score with that of their corresponding H&Y stage. Our findings indicate that the gait variables considered in isolation have a moderate ability to discriminate pwPD from HS, with the best discriminative ability found in gait speed, but their discriminative power increases when they are collected together in the factor score or in the model. Further, when considering pwPD stratified by H&Y stage, it emerges that multiple impairment signs appear at stage 2.5, while at the earlier stages of disease both the asymmetry variables and the asymmetry factor highlight differences between patients and HS.

### Accuracy of the model in differentiating PD from HS

The gait features included in this study were selected according to a validated model of gait. This was created from a sample of pwPD with H&Y stage ranging from 1 to 4. We found that, by using only 8 gait characteristics from three independent gait domains (pace/rhythm, variability and asymmetry), we were able to achieve a good identification of gait impairment in PD. The accuracy of the model is higher than that of the single variables. Previous studies have tried to describe gait of pwPD using different methods, such as vertical ground reaction force^55^, inertial measurement units^56^, tri-axial accelerometer^41^ and instrumented insoles^22^. All these studies were based on a small number of patients, did not take into account the entire spectrum of disease severity, and extracted numerous variables that are not always easily interpretable from a clinical point of view. Besides, the use of wearable technologies does not seem to increase the ability to differentiate the pwPD and HS correctly based on the data simply collected from sensorized mats^57^.

Recently, Rehman et al.^58^ assessed gait of more than 100 pwPD with a GAITRite® walkway and, using a machine learning approach, tried to highlight the group of spatiotemporal variables that best differentiated pwPD from HS. They identified six variables (step velocity, step length, step time, step length variability, step width and step width variability) that reached an accuracy ranging between 73 and 97%. Compared to our study, the main differences are the lack of asymmetry variables in Rehman et al.’s work^58^ and the absence of variables related to step width in our model. We do not believe that “step width” is of major clinical importance to warrant its inclusion in the model due to: (a) the low correlation values of step width with the other variables, as also in Rehman et al.^58^, (b) its low discriminative ability in discriminating pwPD from HS in our dataset where the AUC was 0.60 (not shown in results), and (c) the absence of significant differences in the mean step width between pwPD and HS, as in Roemmich et al.^59^ and as recently stated in a meta-analysis^21^. Conversely, asymmetry is an essential feature in the evaluation of PD gait, since alterations in step time asymmetry are associated with a shorter time to diagnosis^19^ and bear high relevance for characterising de novo pwPD^60^.

Nevertheless, three of the 6 variables identified by Rehman et al.^58^ are the same as those included in our model, which may explain the similar value of accuracy. Of note, both studies seem to consolidate the importance of assessing the variability domain in addition to the pace/rhythm variables. In fact, our findings underline that, among the factors, variability has the greatest accuracy in differentiating pwPD from HS.

#### Pace/rhythm

Gait speed, step time and double support time, belonging to the pace/rhythm factor, showed differences between pwPD and HS which became greater as the disease progressed. The difference is not significant in the first few H&Y stages, but grows from large to huge between stages 2.5 and 4. Gait speed, which is the most frequently reported variable in the studies investigating gait of pwPD^10,21^, appeared to be one of best variables for discriminating gait of PD from HS. Nevertheless, the main difference in gait speed between these two populations appears from stage 2.5 onwards. These results support previous findings based on a large cohort of HS according to which gait speed is not a significant predictor of PD conversion^19^ and are in line with other studies that reported no difference in the mean gait speed of patients with early PD compared to HS^61–63^. Recently, Keloth et al.^64^ also noted that the double-support interval increased with the severity of PD, but the increase was modest and not significant at stage 3. This is in line with our results, since we found medium differences at stages 2.5 and 3, while huge differences were found at stage 4.

With respect to the above-mentioned variables of pace/rhythm factor, step time had the lowest ability to discriminate pwPD from HS. In fact, only patients with more advanced stages of PD showed moderate to large differences in step time compared to HS. This result is not surprising given the numerous studies showing that gait cadence is not usually altered in PD^65–67^ or increased as a compensatory mechanism for the step length reduction^65,66^. More in general, pace/rhythm factor showed the lowest ability to differentiate pwPD from HS compared to the other factors and to the model. The sensitivity of pace/rhythm factor in assessing disease progression became large only on reaching H&Y stage 3.

#### Variability

Gait abnormalities in pwPD include increased stride-to-stride variability, a fluctuation in the value of spatial or temporal variables, or both from one stride to the next^23^. Variability is a reliable marker of gait^9,68,69^, and it shows a similar reliability in pwPD and HS^70^. The variability factor has a good discriminative ability between pwPD and HS and increases with the progression of the disease^14^. Overall, variability compared to pace/rhythm factor shows a stronger difference between pwPD and HS than pace/rhythm from stages 2 to 3. In detail, there were only small differences between pwPD and HS in the CVs at H&Y stages ≤ 2, but at stage 2.5 moderate differences emerged in the CV of step velocity and large differences in the CV of swing time and step length.

Further, the model shows that, in addition to gait speed, the variability of swing time and step length are the first variables in the progression of the disease that crucially show a large difference compared to HS when pwPD reach stage 2.5. A huge difference compared to HS is obvious in patients at stage 4, showing that variability increases progressively as the condition deteriorates. It has been proposed that gait speed and gait variability are linked to different cortical networks^71^ which probably have a different action on the brain stem nuclei controlling gait and whose progressive deterioration may have different tempos.

#### Asymmetry

In PD, there is substantial asymmetry of clinical signs^72^. Usually, asymmetry is present from the onset of the disease and may persist in the more severe cases^73^. Gait asymmetry in pwPD is connected to uncoordinated activity in the leg muscles^26^, is normally present in the lower limbs^74^, but can affect the upper limbs as well^75^. We found that the two markers of temporal asymmetry (step time and swing time asymmetry), collapsed together in the asymmetry factor, had moderate ability to identify pwPD from HS, not superior to the variability factor. Nevertheless, it is interesting to note that both the swing time and, in particular, the step time asymmetry are an early sign of the disease. This is in keeping with the conclusion of Del Din et al.^19^, who posited that higher step time variability and gait asymmetry may allow an earlier diagnosis of PD. In particular, they found that step time asymmetry, which in our study was the most sensitive single measure in the early stages of PD, strongly predicts the risk of PD.

In early PD, the difference with respect to healthy gait for both step time and swing time asymmetry, while not large, is here significant. This difference noticeably diminishes with the progression of the disease, and becomes non-significant at H&Y stage 2, when the signs of the disease became bilateral. However, our analysis highlights a definite increase in the temporal gait asymmetries of pwPD compared to healthy gait also at stages 2.5, 3 and, in particular, a large difference at H&Y stage 4, indicating that asymmetry is not only a marker of early PD, but a major sign accompanying the progress of gait impairment and possibly predisposing to freezing of gait^74^.

Interestingly, this increase in temporal gait asymmetries related to the disease progression is underscored by the asymmetry factor and by the model. Thus, our results strongly support the use of factors in addition to the single gait measures, even if not superior to the step time asymmetry. An accurate assessment of asymmetry would thus appear important in clinical practice, since this aspect may be successfully treated with a specific rehabilitation intervention^76^, even if some authors have found that only spatial but not temporal asymmetry improves with rehabilitation in pwPD at stage 1 to 3^77^.

### Clinimetric value of the modified H&Y scale

The original H&Y scale was the first rating scale to describe the progression of PD^78^, providing an overall indication of the stage of disease and progression of symptoms and disability^79,80^. It correlates with dopaminergic loss and with other scales targeting motor abnormality, disability, quality of life, and has a strong clinimetric performance for motor assessment^43,79,81^. Originally created with 5 levels, the modified version added two intermediate stages, 1.5 and 2.5, to better capture the progression of PD, and this version has been adopted by many researchers^15,45,82^.

With our model, it is possible to establish how powerful a certain gait marker is (be it a variable or factor of the model, or the model itself as a whole) in assessing the extent of the differences between HS and pwPD at each stage. From our results, the addition of the intermediate stage 2.5 in the modified H&Y scale seems more than justified. The differences between pwPD and HS are similar at stages 1 and 1.5, showing that gait worsening does not become significant during these early phases of PD^83,84^. Factor scores and most of the variables of pwPD begin diverging clearly from those of HS only between stages 2 and 2.5. Recent findings show that dopaminergic loss and deficits in gait and postural control occur between the H&Y stages 2 and 3^85^. The modified H&Y scale with the 2.5 stage gives the opportunity of a finer view at a critical point, because the transition from H&Y 2 to 3 marks a pivotal milestone in the progression of PD, when gait and balance disorders cause disability in many everyday activities^86^. In fact, our study shows that the critical changes appear between stages 2 and 2.5 rather than between 2 and 3.

Finally, the increase in the factor scores and in the score of the model was disproportionate in pwPD at stage 4, and this strongly reflects the aggravation of locomotion as the disease worsens. Of note, the worsening of the gait variables in pwPD at stage 4 was not attributable to the appearance of freezing of gait since the trials in which our patients showed episodes of freezing of gait on the electronic walkway were not considered in the analysis.

### Limitations

In this cross-sectional study the distribution of pwPD across the severity stages was not even since most of our patients suffered from mild to moderate PD. However, a sizeable number of less and more severely affected patients was contained in our cohort. In particular, the patients at stage 4 were not so numerous since many of them could not walk without assistance, while the selection criteria excluded all pwPD at H&Y stage 5. Spatiotemporal parameters and gait variability were not stratified across age groups in the healthy subject cohort. Analysis of the magnitude of the ES between pwPD and HS was not done for matched ages, because the primary criterion was the H&Y stage. However, a post-hoc appraisal showed that the pwPD within each stage had a mean age not different from that of the entire HS cohort, except for stage 1 and 1.5. In this regard, a recent article showed that gait-related changes with respect to age appear to be significant in HS but not in pwPD^87^. This limitation is mitigated by the demonstration that gait variability measures remain stable over time and are not influenced by age in HS^88^.

## Conclusions

Efforts to regularly update classification on PD gait, including data on critical markers of disability, are important for evidence-based care planning and resource allocation. This cross-sectional study, based on a large cohort of pwPD, showed that our model holds when applied to a mixed population of pwPD and HS and that the accuracy to differentiate PD from HS increases by grouping the variables into factors and into a model. The asymmetry factor and the model, compared to the single variables, highlight gait differences between pwPD and HS at earlier stages of PD. Further, the model emphasizes significant deviations from a monotonous trend in the gait variables, meaning that not all spatiotemporal variables evolve concurrently. For example, changes in step asymmetry are markers of early PD, while changes in double-support time and swing time variability obtrude at H&Y stage 2–2.5. Overall, the data suggest that the gait pattern worsens in the course of the disease, as more and more brain cells die and the neuronal networks lose more and more capacities^89^, but this progression is definitely different for the individual variables, conveying the suggestion that separate neural networks selectively control distinct gait processes. The asymmetry factor is the strongest predictor of PD even when the variability and pace/rhythm factors hardly help. Conversely, there is no clear-cut threshold in the modified H&Y scale, beyond which gait variables would show abnormal values.

The scores of the variables, factors and model clearly separate the gait features of pwPD and HS, so that the model can be safely used to compare gait between the two cohorts. Although some differences between PD and healthy gait emerges for each of the modified H&Y stages, they become stronger for patients in the upper levels of the scale. Further, our findings support the clinimetric value of the modified H&Y scale that includes 0.5 increments, because several variables start showing a difference compared to HS at 2.5 points of the H&Y scale, and the magnitude of these differences changes at 3.0. The model and its factors are appropriate to assess the complexity of the gait impairment in pwPD, and identify the spatiotemporal variables that become abnormal at a certain stage of the modified H&Y scale. Any improvement in our knowledge of the nature and aggravation of gait quality in pwPD is welcome for the clinician, in the view of selecting the best treatment options^90,91^ and improving the differential diagnosis from other neurodegenerative diseases^92^. Recently, Rennie et al.^93^ demonstrated that a highly challenging balance and gait training significantly impacts pace and rhythm aspects of gait in pwPD. The assessment of different gait domains helps frame the effects of rehabilitation in a coherent context and might help identify the best treatment at different stages of disease.

The results obtained in this study are comparable with and extend those yielded by the preliminary validation study^38,42^, thus showing that the present model is a valid instrument to check gait impairment and their correspondence with severity in PD. The simple application of the scheme describing the most relevant structural features of gait in PD makes the model an attractive and useful tool for clinical practice and research. Future investigations have to assess if the model is valid in longitudinal studies^17^ and confirm that the model not only faithfully represents the commonest pattern of progression in the PD walking disability, but also helps stage individual patients with PD and fine-tune the treatment based on these gait markers.

## Supplementary Information

Supplementary Information.

## Data Availability

The datasets generated during and/or analysed during the current study are available from the corresponding author (I.A.) on reasonable request.
