# Supplementary material for: A pathophysiological model of gait captures the details of the impairment of pace/rhythm, variability and asymmetry in Parkinsonian patients at distinct stages of the disease
Source: Sci Rep. 2021 Oct 27;11:21143. doi: 10.1038/s41598-021-00543-9 (PMC8551236; doi:10.1038/s41598-021-00543-9)
Supplement: Supplementary file 1 — Supplementary Information. [file 41598_2021_543_MOESM1_ESM.pdf]

Supplementary material

Exploratory Factor Analysis (EFA)

The EFA results on the correlation of each variable with the three factors are reported below in Table 1S. The 1st factor, named pace/rhythm, included three variables (gait speed, step time, and double support time), the 2nd variable, named variability, four variables (step velocity CV, step length CV, swing time CV and gait speed), while the 3rd factor, asymmetry, contained two variables (step time asymmetry and swing time asymmetry). The gait speed variable showed significant correlation coefficients in two factors (Table 1S), suggesting that it is representative of the pace/rhythm and, to a lesser extent, of the variability factor.

**Variables**	**Correlation coefficients**		
	**1st Factor**	**2nd Factor**	**3rd Factor**
Gait speed	**-0.609**	**-0.508**	-0.216
Step time	**0.892**	0.172	0.172
Double support time	**0.852**	0.376	0.198
Step velocity CV	0.242	**0.826**	0.148
Step length CV	0.382	**0.642**	0.155
Swing time CV	0.343	**0.764**	0.191
Step time asymmetry	0.223	0.260	**0.688**
Swing time asymmetry	0.298	0.144	**0.685**

Supplementary Table S1. Factor matrix reporting the EFA correlation coefficients between variables and factors. Significant correlation coefficients (>0.50) are reported in bold.

Factor scores

A factor score is a numerical value that indicates a person's relative score on a latent factor. In our manuscript, once EFA was conducted, we computed the factor scores, one for each factor of the gait model. Factor scores can be calculated with two different methods: the regression scoring of Thomson (1951) and the weighted sum score (WSS) method (DiStefano et al., 2009). The first method can be easily calculated after running EFA using the STATA command “predict”. In the second method, described here and performed in our study, factor scores are derived from a weighted sum of factor’s items (i.e. gait variables).

The weighted sum score method

First, the eight variables included in the model of Arcolin et al. (2019) were normalized by a z-score transformation, computed by subtracting the mean value of the variable from each data value and dividing the difference by the SD of the mean of the entire sample, which includes both the HS (normal gait) and pwPD (more impaired gait) [i.e., "gait speed z-score = (gait speed – mean gait speed) / SD gait speed"] (Ware et al., 1994).
Secondly, the factor score of each factor of the model (pace/rhythm, variability and asymmetry) was calculated performing a weighted sum of the z-transformed variables belonging to each factor. In particular, each z-transformed variable belonging to a specific factor was multiplied with its coefficient resulting from EFA (i.e. the correlation coefficient between each variable and factor, see Table 1S), and the products were summed.

Below are the formula used for each factor score:

- *Pace/rhythm factor score$_{wss}$ = (gait speed * -0.609) + (step time * 0.892) + (double support time * 0.852)*
- *Variability factor score$_{wss}$ = (step velocity CV * 0.826) + (step length CV * 0.642) + (swing time CV * 0.764) + (gait speed * -0.508)*
- *Asymmetry factor score$_{wss}$ = (step time asymmetry * 0.688) + (swing time asymmetry * 0.685)*

As a result of these formulae, the HS mean values of factor scores were negative, while in the pwPD, as the disease progressed, the mean values of the factor scores increased, although differently among the three factors. That is because the factor scores were calculated from the z-scores of the gait variables, where the formula contained the variable's mean value of both HS and pwPD.

Variables and factor scores split by H&Y stage in comparison to HS

Supplementary Figure S1 shows the three factor scores of HS and pwPD sorted into H&Y stage. HS mean values were negative, because the factor scores were calculated from the z-scores of the gait variables, where the formula contained the mean value of each variable for the entire sample (HS + pwPD combined). As a consequence, since the HS data were below the mean value of the entire sample, the z-scores of the variables were negative, therefore also the factor scores. In pwPD, with the progression of the disease, the mean values of the factor scores increased, although differently among the three factors. For H&Y stages 1, 1.5 and 2, there was no difference between pwPD and HS in the three factor scores, except for the asymmetry factor at stage 1.5 ($p<0.05$). Conversely, for H&Y stage 2.5 and higher, the factor scores of pwPD and HS became significantly different.

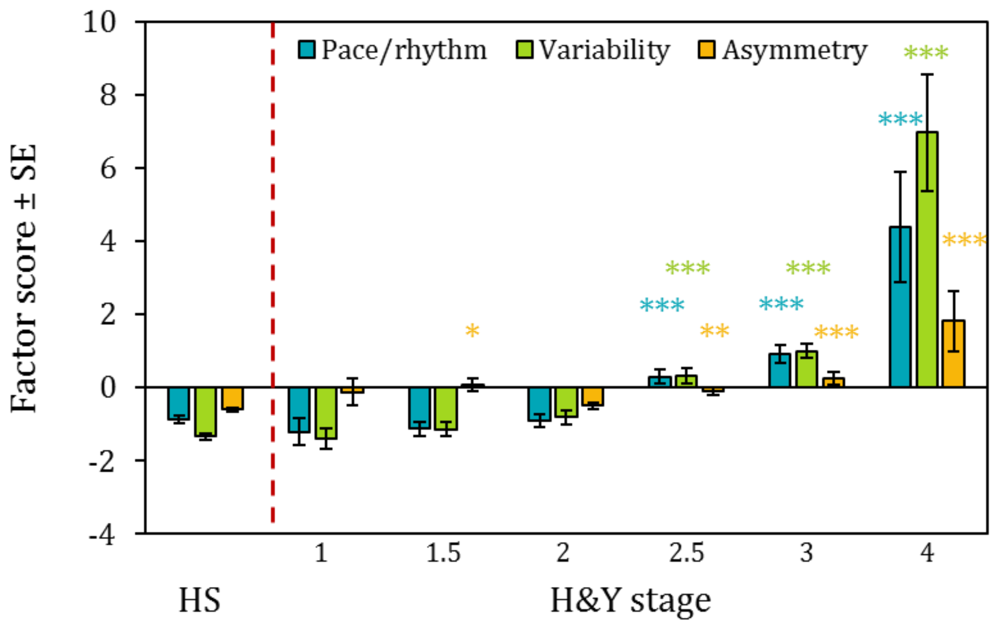

Supplementary Figure S1. Mean values (±SE) of the three factor scores of the HS and pwPD at different stages of the modified H&Y scale. Asterisks represent significant differences between each factor score of HS compared to those of pwPD at different stages: *, $p<0.05$; **, $p<0.005$; ***, $p<0.0005$.

References

Arcolin, I., *et al*. Proposal of a new conceptual gait model for patients with Parkinson's disease based on factor analysis. *Biomed. Eng. Online* **18**, 70; doi:10.1186/s12938-019-0689-3 (2019).

DiStefano, C., Zhu, M., & Mindrila, D. Understanding and using factor scores: Considerations for the applied researcher. *Pract. Assess. Res. Evaluation* **14**, 20 (2009).

Thomson, G. H. The Factorial Analysis of Human Ability. (University of London Press, 1951).

Ware, J. E., Kosinski, M. & Keller, S. D. *SF-36 physical and mental component summary measures: a user's manual*. 4:1-4:4 (Health Assessment Lab, 1994).
